# CD133/Src Axis Mediates Tumor Initiating Property and Epithelial-Mesenchymal Transition of Head and Neck Cancer

**DOI:** 10.1371/journal.pone.0028053

**Published:** 2011-11-28

**Authors:** Yu-Syuan Chen, Meng-Ju Wu, Chih-Yang Huang, Shu-Chun Lin, Tsung-Hsien Chuang, Cheng-Chia Yu, Jeng-Fan Lo

**Affiliations:** 1 Institute of Oral Biology, National Yang-Ming University, Taipei, Taiwan; 2 Graduate Institute of Chinese Medical Science and Institute of Medical Science, China Medical University, Taichung, Taiwan; 3 Institute of Basic Medical Science, China Medical University, Taichung, Taiwan; 4 Department of Health and Nutrition Biotechnology, Asia University, Taichung, Taiwan; 5 Immunology Research Center, National Health Research Institutes, Zhunan, Miaoli County, Taiwan; 6 Institute of Oral Biology and Biomaterial Science, Chung Shan Medical University, Taichung, Taiwan; 7 School of Dentistry, Chung Shan Medical University, Taichung, Taiwan; 8 Department of Dentistry, Chung Shan Medical University Hospital, Taichung, Taiwan; 9 Department of Dentistry, National Yang-Ming University, Taipei, Taiwan; 10 Department of Dentistry, Taipei Veterans General Hospital, Taipei, Taiwan; Virginia Commonwealth University, United States of America

## Abstract

**Background:**

Head and Neck squamous cell carcinoma (HNSCC) is a human lethal cancer with clinical, pathological, phenotypical and biological heterogeneity. Caner initiating cells (CICs), which are responsible for tumor growth and coupled with gain of epithelial-mesenchymal transition (EMT), have been identified. Previously, we enriched a subpopulation of head and neck cancer initiating cells (HN-CICs) with up-regulation of CD133 and enhancement of EMT. Others demonstrate that Src kinase interacts with and phosphorylates the cytoplasmic domain of CD133. However, the physiological function of CD133/Src signaling in HNSCCs has not been uncovered.

**Methodology/Principal Finding:**

Herein, we determined the critical role of CD133/Src axis modulating stemness, EMT and tumorigenicity of HNSCC and HN-CICs. Initially, down-regulation of CD133 significantly reduced the self-renewal ability and expression of stemness genes, and promoted the differentiation and apoptotic capability of HN-CICs. Additionally, knockdown of CD133 in HN-CICs also lessened both *in vitro* malignant properties including cell migration/cell invasiveness/anchorage independent growth, and *in vivo* tumor growth by nude mice xenotransplantation assay. In opposite, overexpression of CD133 enhanced the stemness properties and tumorigenic ability of HNSCCs. Lastly, up-regulation of CD133 increased phosphorylation of Src coupled with EMT transformation in HNSCCs, on the contrary, silence of CD133 or treatment of Src inhibitor inversely abrogated above phenotypic effects, which were induced by CD133 up-regulation in HNSCCs or HN-CICs.

**Conclusion/Significance:**

Our results suggested that CD133/Src signaling is a regulatory switch to gain of EMT and of stemness properties in HNSCC. Finally, CD133/Src axis might be a potential therapeutic target for HNSCC by eliminating HN-CICs.

## Introduction

Head and neck squamous cell carcinoma (HNSCC) is one of the most common cancers in the world and one of causes of cancer-related death due to therapy resistance [1]. Despite of improvements in the diagnosis and treatment of HNSCC, the overall long-term survival rates have improved marginally over the past decade [2].

Accumulating data demonstrate that tumor formation is driven by a subpopulation of cells that exhibit self-renewal capacity–the purported cancer stem cells (CSCs) or cancer initiating cells (CICs) [3], [4]. CICs have been shown to have the capacity to promote tumor progression and metastasis, and also contribute to radio-resistance and chemo-resistance [5]. Recently, we and others have verified the existence of CICs in HNSCC (HN-CICs) [6], [7], [8], [9]. However, there is lack of evidence how cell surface signaling modulating the intracellular stemness properties or tumorigenicity of HN-CICs.

CD133 (prominin-1), a 5-transmembrane glycoprotein, was originally recognized as a hematopoietic stem cells marker [10]. Consequently, CD133 has been considered as an important cell surface marker to represent the subpopulation of CICs in brain tumors, colon carcinoma, prostate carcinoma, hepatocellular carcinoma, thyroid carcinoma and head and neck cancer [8], [11], [12], [13], [14], [15], [16], [17], [18]. Previously, we have demonstrated that the up regulation of CD133 in HN-CICs, further, the up-regulation of C133 in HNSCC cancerous tissue is negatively correlated with the survival prognosis of HNSCC patients [8]. Recent reports also suggest that expression of CD133 in tumor tissues could serve as a prognostic indicator for tumor re-growth, malignant progression, and patient survival [19], [20], [21]. Nevertheless, the CD133 mediated molecular mechanisms in regulating CICs in HNSCC is still unclear.

EMT, a cellular transformation that converts adherent epithelial cells into migratory mesenchymal cells, is critical for embryonic development and tumorigenic progression of cancer cells and cancer metastasis [22]. Researchers have shown that EMT could promote stem cells (SCs) properties and further generate cells with the features of tumor initiating property [23], [24]. EMT program also significantly maintained tumor initiating cells property in HNSCC [6], [7], [25].

Src, a classical non-receptor tyrosine kinase with the potential to cause cell transformation including uncontrolled proliferation and loss of contact inhibition, becomes activated by interacting with the stimulated membrane receptors [26]. The extracellular signal would be further amplified and transduced through interaction between activated Src and downstream targets such as Ras/MAPK, PI3K/AKT and STAT3 pathways [26]. Activation of Src disrupts cell-cell junction, promotes invasiveness through the phosphorylation of β-catenin thus causing the degradation of E-cadherin, and subsequently triggers the EMT [27]. Further, Boivin et al demonstrate that CD133 is a novel binding partner of Src and is phosporylated by Src kinase [28].

The detailed molecular mechanisms involved in the regulatory links between EMT and stem cell–related genes such as CD133 and Src are still poorly understood. Herein, we demonstrate a critical role of CD133 in the enhancement of stemness, gain of EMT, and promoting tumorigenicity of HN-CICs. Additionally, down-regulation of CD133 or inhibition of CD133 induced Src activation lessens stemness properties and tumorigenicity of HNSCCs both *in vitro* and *in vivo.* Ultimately, we demonstrate the significance of CD133/Src signaling on EMT process in HNSCC.

## Materials and Methods

### Cell lines cultivation and enrichment of HN-CICs from HNSCCs

Two HNSCC cell lines, SAS [29] and OECM1[30], were grown in DMEM or in RPMI supplemented with 10% FBS (Grand Island, NY), respectively. One primary HNSCC cell line was obtained from HNSCC patient. All of the clinical samples in this study were approved and in accordance with the Institutional Review Board of Chung Shan Medical University Hospital (CSMUH No: CSI0249). For enrichment of HN-CICs, the two cell lines were cultured in tumor sphere medium consisting of serum-free DMEM/F12 medium (GIBCO), N2 supplement (GIBCO), 10 ng/mL human recombinant basic fibroblast growth factor-basic (FGF) and 10 ng/mL Epidermal Growth Factor (EGF) (R&D Systems, Minneapolis, MN). Cells were plated at a density of 7.5×10^4^ to 1×10^5^ live cells/10-mm dishes, and the medium was changed every other day until the tumor sphere formation was observed in about 4 weeks [8].

### Stable overexpression of CD133 in HNSCC cells

**Figure 1:**
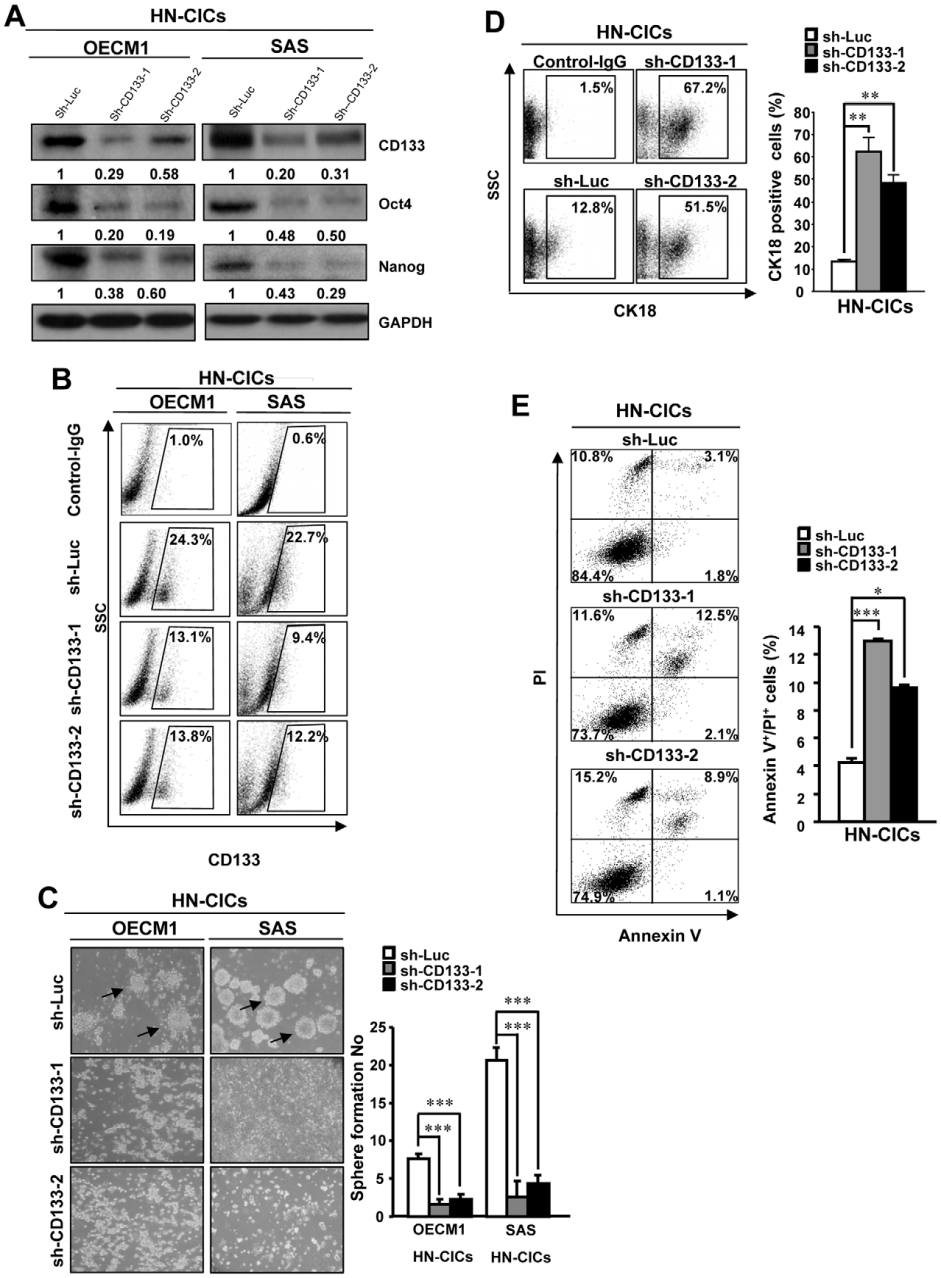
Depletion of CD133 impairs self-renewal property but inversely increases differentiation and apoptotic activity of HN-CICs. (A) Single cell suspension of HN-CICs was transduced with sh-Luc or sh-CD133 lentivirus for 3 days. Total proteins prepared from infected cells were prepared and analyzed. The silencing effect of CD133 shRNA in HN-CICs was validated translationally by western blotting (OECM1 (*left panel*) and SAS (*right panel*)). Immunoblotting against anti-Oct-4, anti-Nanog, or anti-GAPDH antibodies was performed as indicated. The amount of GAPDH protein of different crude cell extracts was referred as loading control, and for further quantification. (B) Cell surface CD133 of sh-CD133-1, sh-CD133-2 and sh-Luc HN-CICs was analyzed by flow cytometry (C) HN-CICs were first infected with sh-CD133-1, sh-CD133-2 or sh-Luc lentivirus for 3 days, and then further cultivated under the serum-free defined selection medium. The cellular morphology of HN-CICs treated with sh-Luc or CD133-shRNA lentivirus was examined. Arrows indicated the sphere cells. The expression profile of CK18 (D) or Annexin V vs. PI positive staining (E) of HN-CICs cells infected with sh-CD133-1, sh-CD133-2 or sh-Luc lentivirus was assessed, respectively, by flow cytometry. The percentage of Annexin V^+^/PI^+^ double positive cells was recorded (E; right panel). The control IgG was used to define the baseline signal in (B) and (D). The experiments were repeated three times and the representative results were shown. Results are means ± SD (*, p<0.05; ***, p<0.001).

Human CD133 gene was amplified from human fetal lung and spleen cDNA template obtained from Biosettia Inc. (Cat. No. cDNA-hsa-09; San Diego, CA, USA) and then cloned into pCDH1-MCS1-EF1-copGFP (System Biosciences, Cat. No: CD511A-1; Mountain View, CA, USA). The sequences of oligos used for CD133 PCR amplification are 5′-ACCGTCTAGAATGGCCCTCGTACTCGGCTCCCTGTTGCTG-3′ and 5′- ATCAAAGCTTATTGAAGCTGTTCTGCAGGTGAAGAG- tgcc-3′. Lentivirus production was performed by co-transfection of plasmid DNA mixture with lentivector plus helper plasmids (VSVG and Gag-Pol) into 293T cells (American Type Culture Collection, Manassas, VA) using Lipofectamine 2000 (LF2000, Invitrogen, Calsbad, CA, USA). The lentivirus M.O.I titer is determined by flow cytometry (average of 5×10^4^ and 2×10^5^ TU/ml). To generate the stable cell lines, sub-confluent HNSCC cells were infected with lentivirus in the presence of 8 µg/ml polybrene (Sigma-Aldrich, St Louis, MO, USA). The green fluorescence protein (GFP), which was co-expressed in lentiviral-infected cells, was served as a selection marker to indicate the successfully infected HNSCCs. Stable CD133-overexpressing HNSCC cell lines were further purified by cell sorting with GFP positive cells (Figures 1A and 1B). The pCDH1-MCS1-EF1-copGFP empty vector alone is utilized for experimental control.

### Construction of Lentiviral-mediated RNAi for silencing CD133

The pLV-RNAi vector, which co-expressing GFP protein in infected host cells, was purchased from Biosettia Inc. (Biosettia, San Diego, CA, USA). The method of cloning the double-stranded shRNA sequence is described in the manufacturer's protocol. Lentiviral vectors expressing shRNA that targets human CD133 (oligonucleotide sequence: Sh-CD133-1:5′-AAAAGGACAAGGCGTTCACAGATTTGGATCCAAATCTGTGAACGCCTTGTCC-3′; Sh-CD133-2:5′-AAAAGGATACACCCTACTTACTATTGGATCCAATA GTAAGTAGGGTGTATCC-3′) were synthesized and cloned into pLVRNAi to generate a lentiviral expression vector. Sh-Luc:5′-CCGGACTTACGCTGAGTACTTCGAA CTCGAGTTCGAAGTACTCAGCGTAAGTTTTTTG-3′ was utilized for experimental control. Lentivirus production was performed as above. Stable pLV-RNAi expressed HNSCC cell lines were further purified by cell sorting with GFP positive cells (Figure S1C).

### Side population analysis

For side population analysis, single HNSCC cells suspension at 1×10^6^/ml was prepared in pre-warmed DMEM medium with 2% fetal bovine serum (FBS). Hoechst 33342 dye was then added at a final concentration of 5 µg/ml in the presence or absence of fumitremorgin C (FTC) (10 µM; Sigma, St Louis, MO, USA) and was incubated at 37°C for 90 min with intermittent shaking. The cells were washed with ice-cold HBSS with 2% FBS and centrifuged at 4°C, and re-suspended in the same buffer. Propidium iodide at a final concentration of 2 µg/ml was added for gating viable cells. The Hoechst 33342 dye was excited at 357 nm and its fluorescence was dual-wavelength analyzed (blue, 402–446 nm; red, 650–670 nm). Analyses were done on a FACS Vantage (BD, San Diego, CA, USA) [6].

### Apoptotic Assay

Apoptotic cells were detected with an Annexin V-APC kit (Calbiochem, Darmstadt, Germany) according to manufacturer's guidelines. After staining, the cells incubated with 20 µg/ml propidum iodide (PI) were analyzed by FACS Calibur apparatus (Becton Dickinson, San Diego, CA, USA) [6].

### In vitro cell migration and invasion assay

For transwell migration assays, 2×10^5^ cells were plated into the top chamber of a transwell (Corning, Acton, MA) with a porous membrane (8.0 µm pore size). Cells were plated in medium with lower serum (0.5% FBS), and medium supplemented with higher serum (10% FBS) was used as a chemoattractant in the lower chamber. The cells were incubated for 24 h at 37°C and cells that did not migrate through the pores were removed by a cotton swab. Cells on the lower surface of the membrane were stained with Hoechst 33258 (Sigma-Aldrich) to show the nuclei; fluorescence was detected at a magnification of 100x using a fluorescence microscope (Carl Zeiss, Oberkochen, Germany). The number of fluorescent cells in a total of five randomly selected fields was counted. In vitro cell invasion analysis was described previously [8].

### Soft agar colony forming assay

Each well (35 mm) of a six-well culture dish was coated with 2 ml bottom agar (Sigma-Aldrich) mixture (DMEM, 10% (v/v) FCS, 0.6% (w/v) agar). After the bottom layer was solidified, 2 ml top agar-medium mixture (DMEM, 10% (v/v) FCS, 0.3% (w/v) agar) containing 2×10^4^ cells was added, and the dishes were incubated at 37°C for 4 weeks. Plates were stained with 0.005% Crystal Violet then the colonies were counted. The number of total colonies with a diameter ≥100 µm was counted over five fields per well for a total of 15 fields in triplicate experiments.

### Subcutaneous xenografts in nude mice

All the animal practices in this study were approved and in accordance with the Institutional Animal Care and Use Committee (IACUC) of National Yang-Ming University, Taipei, Taiwan (IACUC approval No. 961230). Stable sh-Luc and sh-CD133 HN-CICs or control GFP and CD133-overexpresiing HNSCCs mixed with Matrigel (BD bioscience, San Diego, CA, USA) (1:1) were injected subcutaneously into BALB/c nude mice (6–8 weeks). Tumor volume (TV) was calculated using the following formula: TV (mm^3^)  =  (Length × Width ^2^)/2.

### Statistical analysis

Statistical Package of Social Sciences software (version 13.0) (SPSS, Inc., Chicago, IL) was used for statistical analysis. Student's *t* test was used to determine statistical significance of the differences between experimental groups; *p* values less than 0.05 were considered statistically significant. The level of statistical significance was set at 0.05 for all tests.

## Results

### Down-regulation of CD133 reduces stemness properties coupled with increased differentiation and apoptotic capabilities in HN-CICs

We have identified a subpopulation of head and neck cancer initiating cells (HN-CICs) with enhanced stemness properties from HNSCC cells (HNSCCs) by sphere formation assay [8]. Additionally, we and others display up-regulation of CD133 in HN-CICs [8], [31], [32]. To further investigate whether CD133 plays a role in maintaining CICs properties of HN-CICs, the approach of loss-of-function of CD133 was first conducted. Down-regulation of CD133 in HN-CICs was achieved by viral transduction with lentiviral vector expressing small hairpin RNA (shRNA) targeting CD133 (sh-CD133-1 and sh-CD133-2), and lentiviral vector expressing shRNA against luciferase (sh-Luc) was used as control. Immunoblotting analyses confirmed that lentivirus expressing both sh-CD133-1 and sh-CD133-2 markedly reduced the expression level of CD133 protein in transduced HN-CICs (Figure 1A). Cell population with surface CD133-positive (CD133^+^) was also decreased in sh-CD133 infected HN-CICs by FACS analyses (Figure 1B). Furthermore, HN-CICs infected with sh-CD133 lentivirus did not maintain floating spheres but showed more attached epithelial-like cells (Figure 1C). We also observed reduced expression of stemness genes (Oct-4 and Nanog) (Figure 1A) but the enhanced expression of epithelial differentiation marker, CK18 (Figure 1D) in CD133 knockdown HN-CICs. To further determine whether the reduction in tumor sphere formation efficiency with CD133 down-regulation was due to decreased HN-CICs survival, we examined the apoptotic cells using Annexin V plus propidium iodide (PI) staining. HN-CICs infected with sh-CD133 expressing lentivirus significantly increased the percentage of Annexin V^+^/PI^+^ cells (Figure 1E). Together, these data further support that down-regulation of CD133 resulted in a reduction of CICs properties and cell viability in HN-CICs.

### Targeting CD133 abrogates in vitro and in vivo malignant properties of HN-CICs

**Figure 2:**
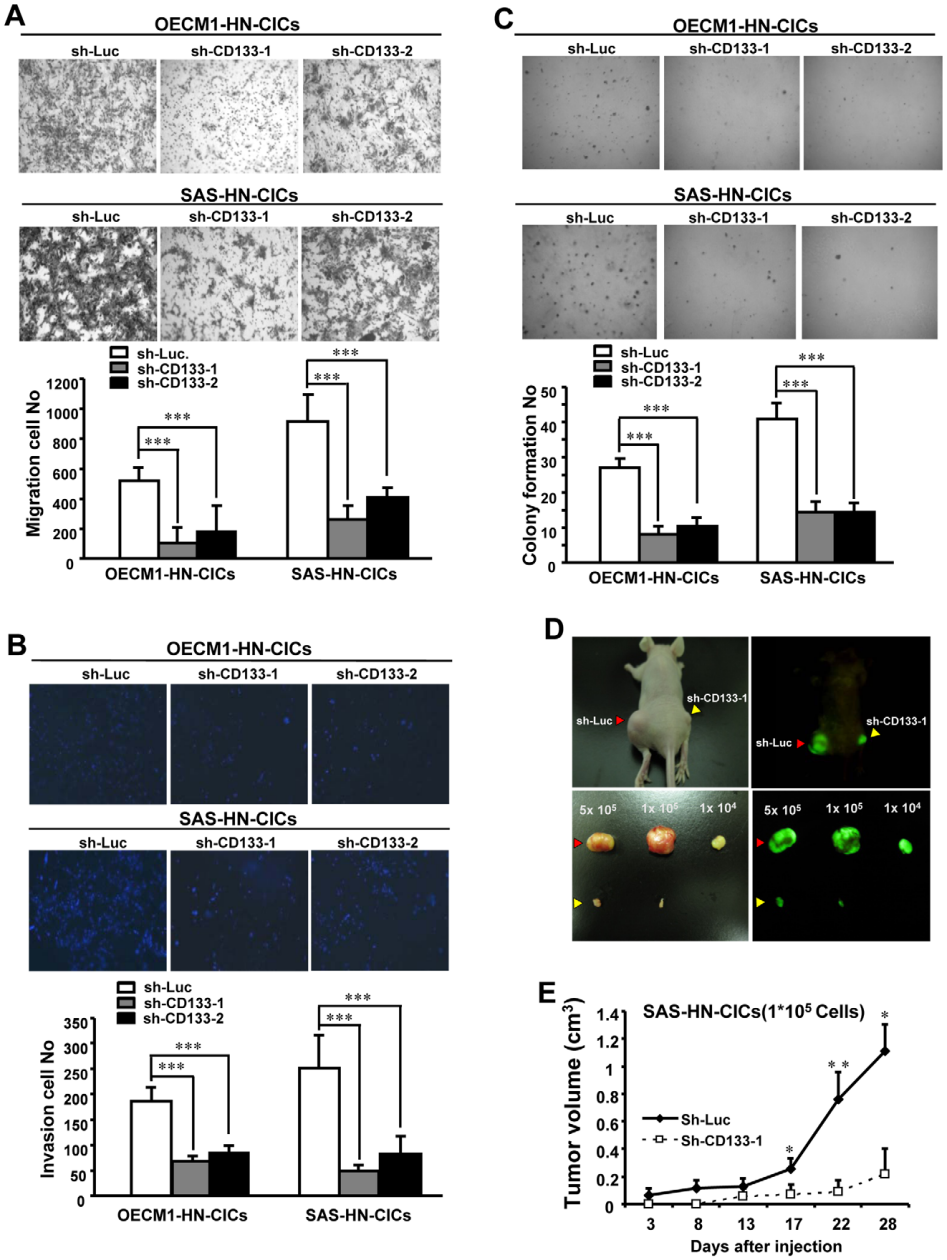
CD133 knockdown reduces migration/invasiveness/clonogenicity and impairs in vivo tumorigenic properties of HN-CICs. To elucidate the capability of cell migration (A), cell invasiveness (B) and anchorage independent growth (C) of HN-CICs (OECM1 (*upper panel*), SAS (*lower panel*)) with CD133 down-regulation, single cell suspension of HN-CICs infected with CD133-specific shRNA or control sh-Luc lentivirus for three days were plated onto transwell, transwell coated with matrigel and soft agar, respectively, and analyzed as described in Materials and Methods. Results are means ± SD of triplicate samples from three experiments. (D) Representative tumors of control HN-CICs and of CD133-knockdown SAS-derived HN-CICs were generated, and the tumors were then dissected from the subcutaneous space of recipient mice (n = 3)(Phase contrast: *left two panels*; GFP imaging*: right two panels*) (Red arrows: sh-Luc HN-CICs; Yellow arrows: sh-CD133-1 HN-CICs). (E) Tumor volume was measured, respectively, after inoculation of CD133-knockdown or sh-Luc–expressing SAS-derived HN-CICs. Error bars correspond to SD. (*, p<0.05; ***, p<0.001)

To elucidate the direct effect of CD133 knockdown on *in vitro* malignant properties including abilities of cell migration, matrigel invasion and anchorage independent growth of HN-CICs, single cell suspension of control- or CD133*-* knockdown HN-CICs were plated onto Transwell chamber (Figure 2A), Transwell chamber coated with matrigel (Figure 2B) or into soft agar (Figure 2C), and analyzed. The migratory/invasion/colony formation abilities of CD133 knockdown HN-CICs were significantly reduced (Figures 2A, 2B and 2C). We next sought to determine if down-regulation of CD133 expression could attenuate the tumorigenic ability of HN-CICs *in vivo*. Of note, inhibition of CD133 expression significantly slowed down the tumor growth mediated by HN-CICs (Figures 2D and 2E; p<0.05; p<0.01). In addition, the immunohistological staining of tumors derived from sh-CD133 HN-CICs displayed decrease of Oct4 and increase of CK18 in comparison to those from control HN-CICs tumors (Figure S2A and S2B). Overall, our data indicate that depletion of CD133 inhibits tumor initiating activity of HN-CICs *in vivo*.

### Overexpression of CD133 enhances stemness properties and tumorigenic potentials of HNSCCs

**Figure 3:**
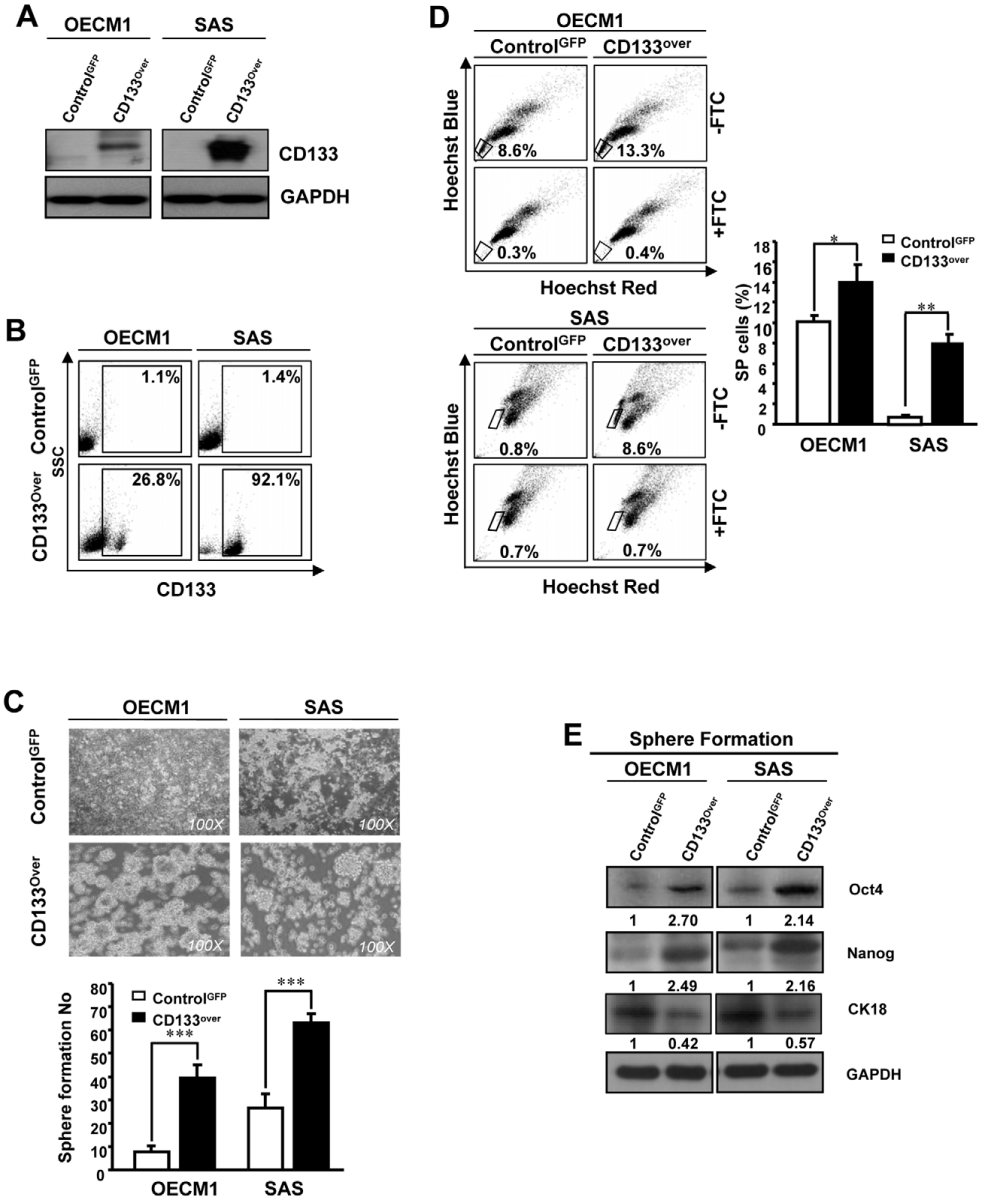
Overexpression of CD133 in HNSSCs promotes stemness properties. (A) Expression of CD133 protein in HNSCCs infected with either CD133-overexpressing or control GFP lentiviruse was examined by western blot. The amount of GAPDH protein was referred as loading control. (B) Cell surface CD133 expression in CD133-overexpressing or control HNSCCs was analyzed by flow cytometry. (C) Representative images of tumor sphere formation ability of control-GFP or CD133-overexpressing HNSCCs. (D) Single cell suspensions of stable CD133-overexpressing and control GFP-expressing HNSCCs were incubated with Hoechst 33342 in the presence or absence of 10 µM fumitremorgin C (FTC), then, analyzed by flow cytometry to identify the SP cells. (E) Total crude cell extracted proteins from control-GFP or CD133-overexpressing HNSCCs under cultivation with defined serum-free medium for 2 weeks were prepared and immunoblotted against anti-Oct-4, anti-Nanog, anti-CK18 or anti-GAPDH antibodies as indicated. The amount of GAPDH protein of different crude cell extracts was referred as loading control for further quantification.

**Figure 4:**
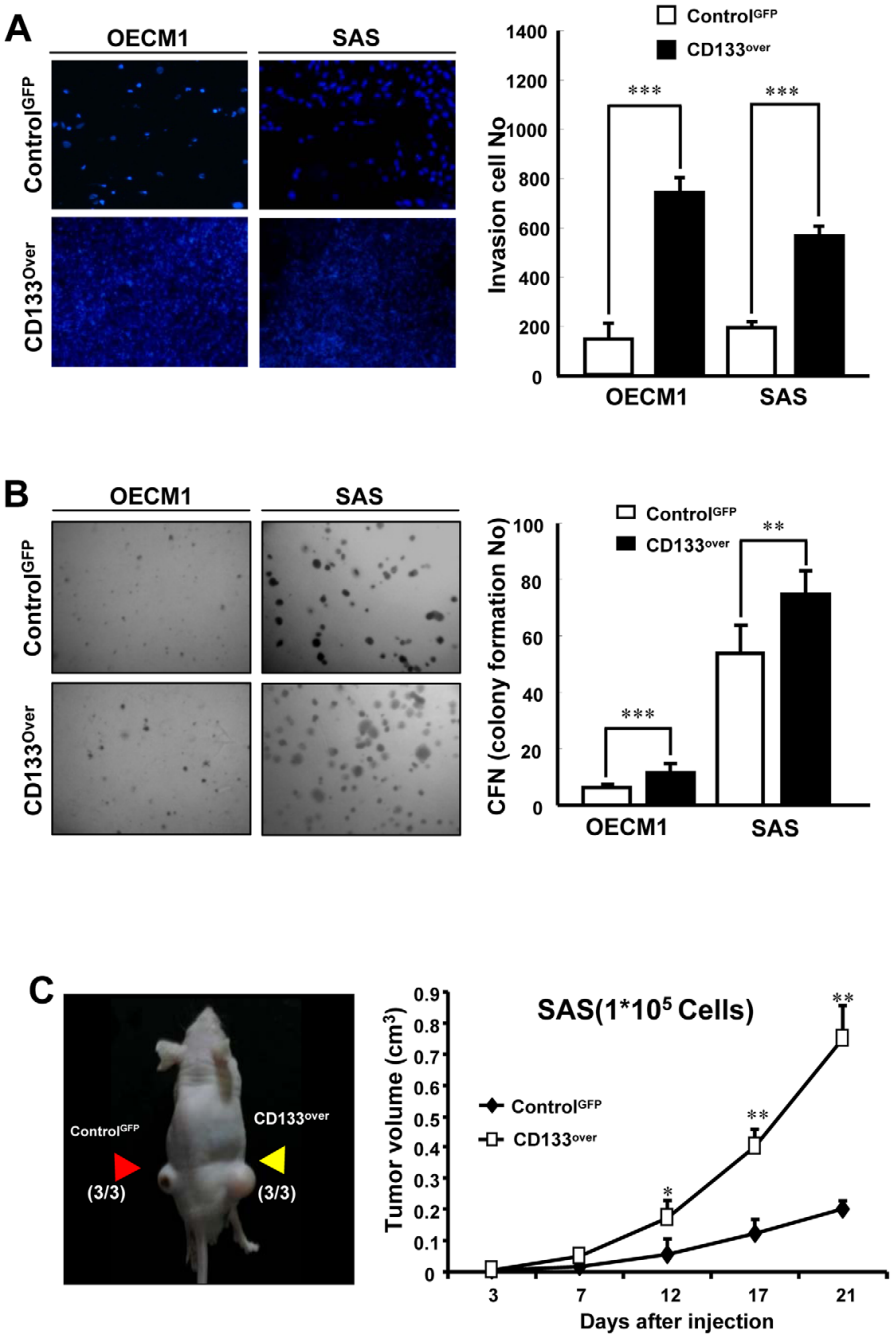
Overexpression of CD133 in HNSCCs enhances malignant activities. (A) The invasiveness ability of control-GFP or CD133-overexpressing HNSCCs were examined as described in materials and methods (*, p<0.05; ***, p<0.001). (B) Anchorage-independent growth of control-GFP or CD133-overexpressing HNSCCs was analyzed (**, p<0.01; ***, p<0.001). (C) Representative tumor growth of control-GFP or CD133-overexpressing HNSCCs (1X10^5^ cells) in the subcutaneous space of recipient mice (Red arrows: control-GFP HNSCCs; Yellow arrows: CD133-overexpressing HNSCCs)(*left panel*). Tumor volume was measured after inoculation of control-GFP (n = 3) or CD133-overexpressing HNSCCs (n = 3), respectively (*right panel*). Error bars correspond to SD (*, p<0.05; **, p<0.01).

To further investigate the effect of CD133 up-regulation on biological properties of HNSCCs, we generated stable CD133-overexpressing HNSCCs through lentiviral-mediated transduction. The successful infection rate of control-GFP and CD133-overexpressing HNSCCs, afterward the cell sorting, ranged from 93 to 94% (OECM1) and 97 to 91% (SAS), respectively (Figures S1A and S1B). The two CD133-overexpressing HNSCCs (OECM1 and SAS) displayed elevated expression of CD133 by western blot analyses (Figure 3A). Further, we found that compared to GFP expressing control cells the CD133-overexpressing HNSCCs showed significant increasing of CD133^+^ cells by FACS analyses (Figure 3B). In addition, the CD133-overexpressing HNSCCs showed enhanced tumor sphere-forming capacity (Figure 3C) and significant increasing of side population (SP) cells (Figure 3D). We also observed that increased protein level of Oct-4 and Nanog of CD133-overexpressing HNSCCs under cultivation with defined serum-free medium (Figure 3E). Next, we demonstrated that overexpression of CD133 also resulted in increased ability on cell invasiveness and colony formation of HNSCCs (Figures 4A and 4B). Of note, CD133-overexpressing HNSCCs also showed significantly elevated tumorigenicity in comparison to control HNSCCs by xenotransplantation analyses *in vivo* (Figures 4C; p<0.05; p<0.01). In addition, IHC analyses demonstrated that tumors derived from CD133-overexpressing SAS cells displayed more Oct4 but less CK18 staining (Figure S2C). Collectively, these results suggest that overexpression of CD133 promotes stemness properties and tumorigenicity of HNSCCs.

### Src kinase is the downstream modulator of CD133 on regulating EMT in HNSCC and HN-CICs

**Figure 5:**
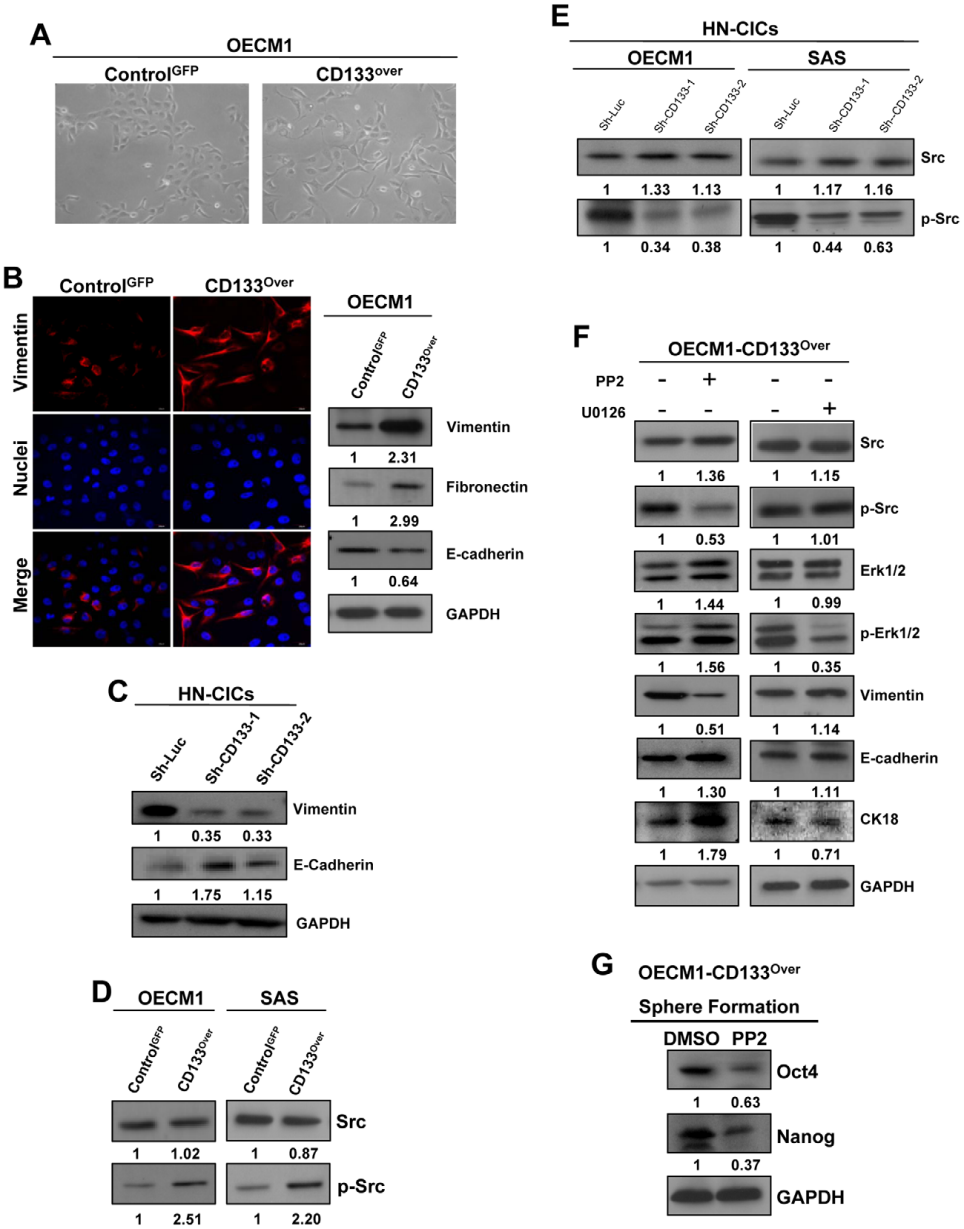
CD133/Src signaling regulates the mesenchymal transformation in HNSCCs and HN-CICs. (A) Morphological difference between control-GFP and CD133-overexpressing HNSCCs. (B) Immunoblotting analysis (*right panel*) and confocal immunofluorescent staining (*left panel*) of EMT-related markers in control-GFP or CD133-overexpressing HNSCCs were analyzed. (C) The protein levels of Vimentin and E-cadherin in the indicated HN-CICs were analyzed by western blot. (D) Protein level of Src or p-Src in control-GFP or CD133-overexpressing HNSCCs were analyzed by immunoblotting. (E) Single cell suspension of HN-CICs was infected with sh-Luc-expressing or shRNAi CD133 lentirus, respectively, and the expression of Src or p-Src in above HN-CICs was analyzed by western blot. (F) CD133-overexpressing HNSCCs were first treated with 10 µM PP2 (Src inhibitor) or 10 µM U0126 (Erk inhibitor) for 24 hours. The expression of Src, p-Src, Erk1/2, p-Erk1/2, vimentin, E-caherin, or CK-18 of above treated cells was evaluated by western blot analysis with GAPDH being an internal loading control. (G) CD133-overexpressing HNSCCs were first cultured with defined serum-free medium for 2 weeks along with the addition of PP2, and the expression of Oct-4, Nanog, or GAPDH proteins in control (DMSO) or PP2 treated cells was analyzed by immunoblotting.

HNSCC epithelial cells can acquire mesenchymal traits which facilitate migration and invasion through EMT process [7], [33]. In figures 4A, we showed that CD133 promotes invasive ability of HNSCCs, we then wanted to explore whether CD133 would modulate the EMT pathway. Morphological observation indicated that overexpression of CD133 in epithelial-type OECM1 cells revealed mesenchymal-like shape change (Figure 5A). Immunofluorescence and immunoblotting staining displayed that an epithelial-like protein expression pattern (E-Cadherin) was decreased but a mesenchymal-like protein expression pattern (Vimentin and Fibronectin) was enhanced in CD133-overexpressing HNSCCs (Figure 5B). On the other hand, silencing of CD133 reduced mesenchymal marker (Vimentin) but induced epithelial markers (E-Cadherin) in HN-CICs (Figure 5C).

Src signaling pathway is an important mediator of the EMT in HNSCC (Figure S3A) [34]. Fujimoto et al have showed that tyrosin-513 within CD19 is not only Lyn (a Src family kinase) binding site but also phosphorylation side, further, the interaction between CD19 and Lyn would amplify the Src family kinase activity and downstream cascade [35]. Boivin et al have demonstrated that CD133 is a novel binding partner of Src and is phosporylated by Src kinase [28]. However, how does the interaction between CD133 and Src kinase activate the downstream effects including amplification of Src activity remain unclear? Here, we demonstrated that p-Src (activated Src with phosphorylation) was increased in CD133-overexpressing HNSCCs (Figure 5D). On the contrary, CD133 silencing reduced active Src in HN-CICs (Figure 5E). Consistently, treatment of Src inhibitor (PP2) but not Erk1/2 inhibitor (U0126) significantly reversed the mesenchymal-like protein expression pattern into an epithelial-like one, and increased the expression of cytokeratin 18 (CK18), a differentiation marker, in CD133-overexpressing OECM1 cells (Figure 5F). Overexpression of CD133 promotes the phosphorylation of Erk1/2 in human glioblastoma cells [36], but in our CD133-overexpressing HNSCCs, treatment of Erk1/2 inhibitor (U0126) caused only slight influence on CD133-overexpressing induced EMT (Figure 5F and data not shown). Lastly, PP2 treatment reduced stemness markers (Oct4 and Nanog) and also impaired sphere formation ability of CD133-overexpressing HNSCCs under cultivation with defined serum-free medium (Figure 5G and data not shown).

### CD133 downregulation and Src inhibition abrogate the p-Src activity and sphere formation ability in primary HN-CICs

**Figure 6:**
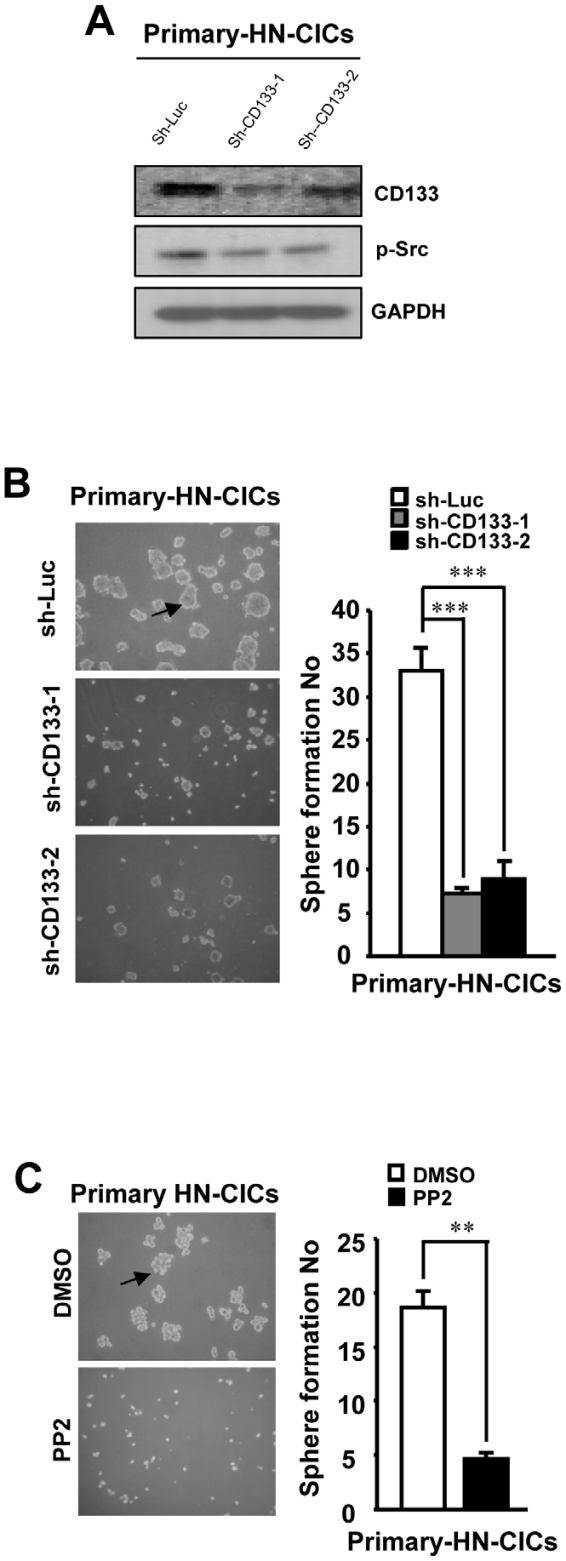
CD133 downregulation and Src inhibition abrogate the p-Src activity and sphere formation ability in primary HN-CICs. (A) Protein level of CD133 and p-Src of lentivius mediated CD133 knockdown primary HN-CICs was analyzed by western blot. (B) Primary HN-CICs were first infected with sh-Luc or CD133-shRNA lentivirus. Three days after the lentiviral infection, the sphere formation ability of virus infected cells then cultivated under selection medium were recorded. (C) Newly enriched primary HN-CICs were treated with PP2 (10 µM) for 72 hrs and the sphere formation ability of PP2 treated HN-CICs cells were examined. Arrows indicated the sphere cells.

To further verify the physiological function of CD133/Src axis mediated signaling in primary HN-CICs, the HN-CICs derived from primary HNSCC patient cells were generated. Further, the expression of CD133 in HN-CICs was downregulated by lentiviral-sh-RNAi. Consistently, the p-Src activity was also downregulated (Figure 6A). In addition, the sphere formation ability of sh-CD133 primary HN-CICs was decreased compared to that of control (sh-Luc) primary HN-CICs (Figure 6B). Finally, PP2 (inhibitor of Src activity) treatment to primary HN-CICs also abolished the sphere formation capability (Figure 6C).

**Figure 7:**
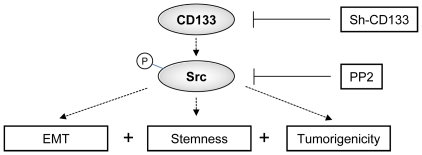
Schematic of the CD133/Src signaling pathways promoting EMT, stemness properties, and tumorigenicity of head and neck cancer initiating cells (HN-CICs). Up-regulation of CD133, consequently, activates the Src signaling (p-Src), then, promotes gain of EMT, enhancement of stemness properties and tumorigenicity in HNSCC.

In Summary, our results suggested that CD133/Src signaling plays a major switch on regulating CICs properties, EMT transformation and tumorigenicity of HNSCCs (Figure 7).

## Discussion

Tumor is composed of a heterogeneous population of cells, and it has been observed that a subpopulation of cells, so called cancer stem cells (CSCs) or cancer initiating cells (CICs), within tumor tissues posses stemness properties [37]. CSCs or CICs are considered to be responsible for the initiation, propagation and metastasis of tumors [38], [39], [40]. Importantly, the existence of CICs might explain cancer recurrences because of radioresistance or chemoresistance after clinical treatment on cancer patients [5].

CD133, a cell surface marker of hematopoietic stem cells and endothelial progenitors, has been proposed to be involved in the angiogenesis as well as cancer tumorigenicity [41]. CD133 has recently been identified as a common CICs marker for many tumor types, including HNSCC [8], [31]. Bao et al. find that the subpopulation of CD133^+^ cells isolated from brain tumors exhibit CICs properties, are refractory to chemo- or radiotherapy, and promote angiogenesis to facilitate the brain tumor growth [42]. Meanwhile, CD133^+^ CICs in solid tumors characterized confer resistance to chemotherapy [43]. On the contrary, CD133 depletion represses colony-forming ability of colon cancer [44]. Therefore, better understanding of the biological characteristics of CD133^+^ CICs may explain the failure of cancer management, and will provide us with new therapeutic approaches [45].

Herein, we evaluated the role of CD133 in the maintenance of stemness characteristics and tumorigenic potential of HNSCCs and HN-CICs by lentiviral-mediated knockdown or overexpression of CD133 (Figures 1 and 3). Depletion of CD133 promoted differentiation and decreased i*n vivo* tumorigenic properties of HN-CICs (Figures 1 and 2). Whereas, overexpression of CD133 enhances tumor sphere-forming capability, side population cells, stemness genes expression (Oct4 and Nanog) and promotes tumorigenic ability of HNSCC (Figures 3 and 4). Collectively, our data first demonstrated the crucial role of CD133 in the stem-like enhancement and tumorigenesis of HNSCC and HN-CICs.

EMT, a de-differentiation program that converts adherent epithelial cells into individual migratory cells, is critical for embryonic development, the oncogenic progression of tumor cells, and cancer metastasis [22]. Enhanced EMT characteristic is associated with poor overall and metastasis-free survival in patients with HNSCC [33]. Single or combined overexpression of stemness factors, including Oct-4 and Nanog, were associated with cancer stem-like properties and EMT [46], [47]. Additionally, activation of Src is frequently associated with human cancer because there is evidence of a prominent role of Src in EMT and development of metastasis. Further, CD133 has been described as a substrate of Src. The identification of CD133 as a substrate for Src suggests that the unknown function of the cytoplasmic domain of C133 might mediate important physiological function in subcellular regulation [28]. In line with these reports, we firstly showed CD133/Src axis regulated tumor initiating property and EMT traits. The discovered roles of stemness signature coupled with EMT process has gained huge interest in the field of cancer research as it indicates that misplaced stemness properties contribute to tumor metastasis and recurrence, making cancer difficult to be tackled. A further understanding on the regulatory networks between EMT and stemness signature may update our current knowledge on the development of therapeutic treatments for malignant cancers in the future.

Hypoxia is a well-recognized tumor microenvironment/niche that is linked to tumor aggressiveness [48], [49], and also favor invasive growth and malignant progression by stimulating the Src pathway. Recently, many reports demonstrated hypoxia is also crucial in maintaining the stem cells and cancer stem cells niche [50], [51]. For example, hypoxia increases SP cells having high tumorigenicity and CICs characteristics including Oct-4 up-regulation [52]. Notably, HIF-1α promotes CD133-positive human glioma-derived CICs propagation and self-renewal [53], [54]. Matsumoto et al elucidated a mechanistic relationship between CD133 and the hypoxia-inducible factor-1α (HIF-1α) [55]. Interestingly, HIF-2α shows strong tumor-promoting activity and has been shown to bind to the Oct-4 promoter for Oct-4 induction in embryonic stem cells [56]. Other studies demonstrated that HIF-2α appears to be an attractive target because it is specifically expressed by brain tumor stem cells but not neural progenitor cells, whereas HIF-1α is up-regulated in these cellular populations [57]. Additionally, Li et al observed HIF-2α co-expressed with CD133 in human glioblastoma biopsy specimens [57]. Our data also found CD133-overexpressing HNSCCs enhanced the expression of HIF-1α or HIF-2α (Data not shown). It is therefore possible that CD133 and HIF might also be the coordinated regulation by hypoxia in HN-CICs. Further research effort is needed in this area.

Extracellular membrane receptor, such as CD19, could interact with and phosphorylated by Src family kinase then amplify the activity of Src family kinases in B lymphocyte [35]. Boivin et al report that there are five tyrosine residues (818, 819, 828, 846, and 852) within CD133 C-terminal domain, which could be potentially phosphorylated by Src kinase. Empirically, the tyrosine-828 residue is the Src SH2 binding motif (YDDV motif)[28]. Here, we tried to explore whether tyrosine phosphorylation in CD133 may promote Src kinase activity. However, we found that CD133Y828F mutant protein did not invert Src activation (Figure S4). It is reasonable that other tyrosine residues of CD133 may be the major regulatory sites on Src activity. Therefore, more precise and sophisticated experimental designs are required to dissect the effect on CD133 up-regulation and tyrosine phosphorylation and consequent activation of Src kinase.

Increased tumor initiating activity is a hallmark of CICs [4]. Knockdown of CD133 lessened tumor initiating activity both *in vitro* and *in vivo* (Figures 1 and 2). However, deletion of CD133 did not completely eliminate tumor promoting potential of HN-CICs (Figure 2E). It is possible that CD133 signaling is among one of the molecular mechanisms in regulation of HN-CICs in HNSCC, although, others have observed that CD133 regulates Notch, Wnt, ERK, and PTEN-PI3K-Akt signaling [15], [58], [59]. Other developmental signaling pathways, including Hedgehog signaling and Bmi1 signaling, have been reported to play critical roles in the regulation of CICs characteristics, which were not significant changed in CD133-knockdown or CD133-overexpression HNSCCs (data not shown). It would be interesting to determine the potential cross-linking of CD133 signaling with other signaling pathways in the future. Overall, our present research showed CD133/Src play a major role in the maintenance of HN-CICs population through EMT modulation. Targeting CD133/Src signaling might be a potential therapeutic target for HNSCC by eliminating CICs.

## Supporting Information

Figure S1 **Establishment of CD133 overexpressing HNSCCs or CD133 stable knockdown HN-CICs.** HNSCC cells, **(**A**)** SAS and **(**B**)** OECM1, were infected with lentivirus co-expressing GFP and CD133. Afterward virus transduction, GFP positive HNSCCs were sorted according to the expression of GFP to isolate stable HNSCCs. GFP positive HNSCC cells demonstrated the successful lentivirus infection. **(**C**)** Single cell of HN-CICs (derived from SAS cells) were infected with lentivirus co-expressing GFP and small hairpin RNA targeting CD133. The successful infected cells were sorted by flow cytometry according to the co-expression of GFP protein.
(TIF)

Figure S2 **Expression of Oct4 and CK18 in tumors derived from CD133 ovexpressing HNSCCs or CD133 stable knockdown HN-CICs.** Tumors derived from control HN-CICs (sh-Luc) or CD133-knockdown SAS-HN-CICs, and SAS cells were collected, sectioned and stained with Hematoxylin and anti-Oct4 (A) or anti-CK18 (B) as described. Arrows indicate the positive staining. (C) Tissue sections of control-GFP and CD133-overexpressing SAS cells xenograft tumor were stained with CK18 or Oct4.
(TIF)

Figure S3 **Src kinase activation promotes EMT, tumorigenicity and stemness.** (A) Expression of p-Src, E-cadherin and Vimentin in HNSCCs transfected with either Src-overexpressing or control vector was examined by western blot. The amount of GAPDH protein was referred as loading control. (B) Anchorage-independent growth of control-vecor or Src-overexpressing HNSCCs was analyzed (**, p<0.01). (C) Sphere formation ability of control or Src-overexpressing HNSCCs was examined under the serum-free defined selection medium (**, p<0.01). (D) Representative tumor growth of control- or Src-overexpressing (Src^over^) HNSCCs in the subcutaneous space of recipient mice (Red arrows: control HNSCCs; Yellow arrows: Src-overexpressing HNSCCs). (E) SAS HN-CICs (5*10^5^ cells) were subcutaneously injected into both backs of nude mice and allowed to develop tumors to a size around 0.2 cm^3^ (12 days). On day 12, 15, and 18 after the inoculation of HN-CIC cells, PP2 (10 µM) was injected into the right back tumors whereas DMSO was injected into the left back as negaive control (Red arrows: DMSO treatment as control; Yellow arrows: PP2 treated HN-CICs).
(TIF)

Figure S4 **Effect of CD133Y828F mutant on p-Src activity.** (A) DNA sequencing confirmed the CD133Y828F mutation, which encodes CD133Y828F mutant protein. (B) 293A cells transfected with plasmids, control-GFP, CD133 wild type (CD133over) or CD133Y828F (CD133Y828F), respectively, were collected (under no serum culture condition). The protein levels of p-Src, total Src and CD133 were examined by immunoblot.
(TIF)
