# Supplementary figures and images for: CD133/Src Axis Mediates Tumor Initiating Property and Epithelial-Mesenchymal Transition of Head and Neck Cancer
Source: PLoS One. 2011 Nov 28;6(11):e28053. doi: 10.1371/journal.pone.0028053 (PMC3225383; doi:10.1371/journal.pone.0028053)

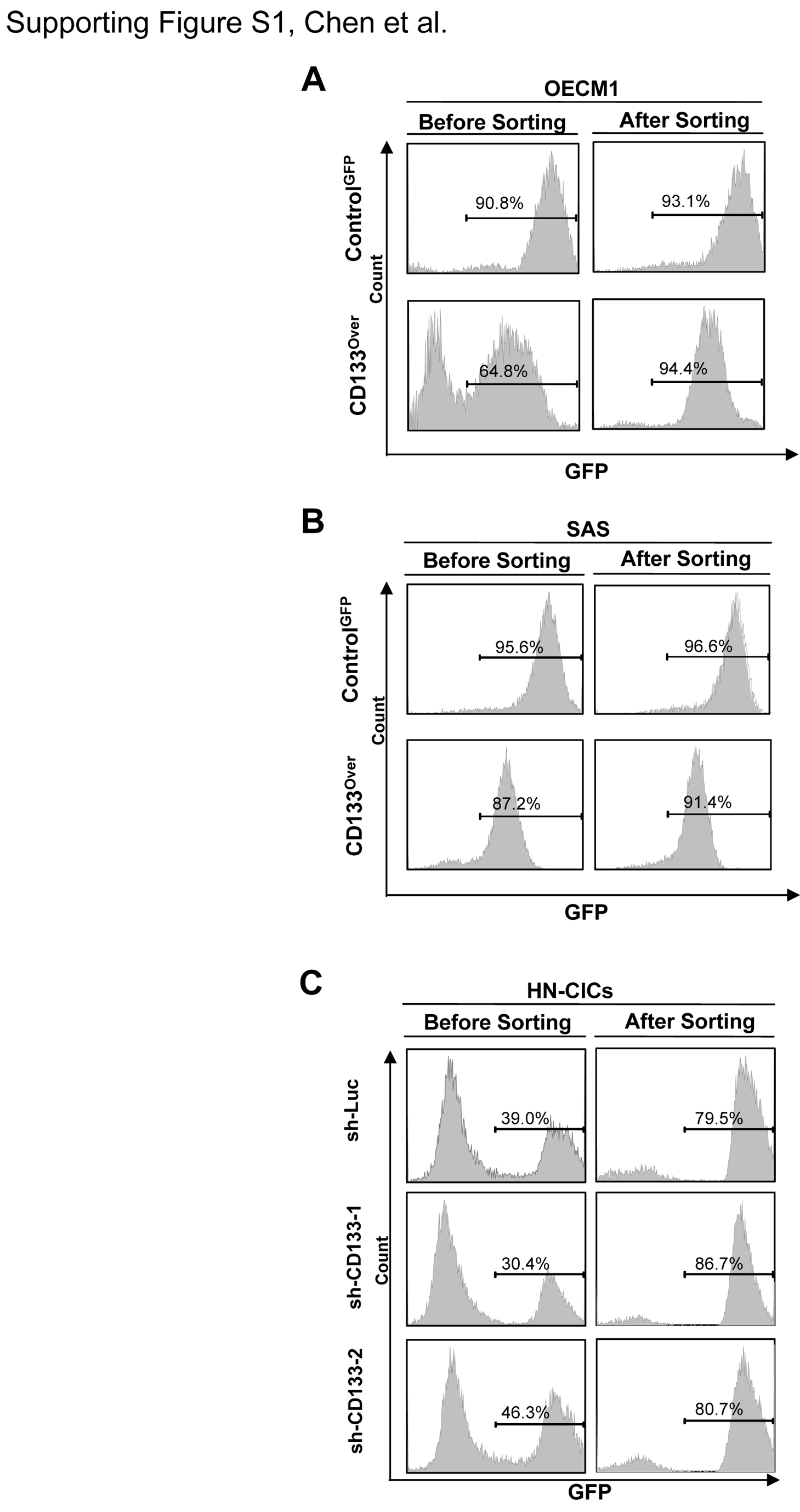

Supplement: Figure S1 — Establishment of CD133 overexpressing HNSCCs or CD133 stable knockdown HN-CICs. HNSCC cells, (A) SAS and (B) OECM1, were infected with lentivirus co-expressing GFP and CD133. Afterward virus transduction, GFP positive HNSCCs were sorted according to the expression of GFP to isolate stable HNSCCs. GFP positive HNSCC cells demonstrated the successful lentivirus infection. (C) Single cell of HN-CICs (derived from SAS cells) were infected with lentivirus co-expressing GFP and small hairpin RNA targeting CD133. The successful infected cells were sorted by flow cytometry according to the co-expression of GFP protein. (TIF) [file pone.0028053.s001.tif]

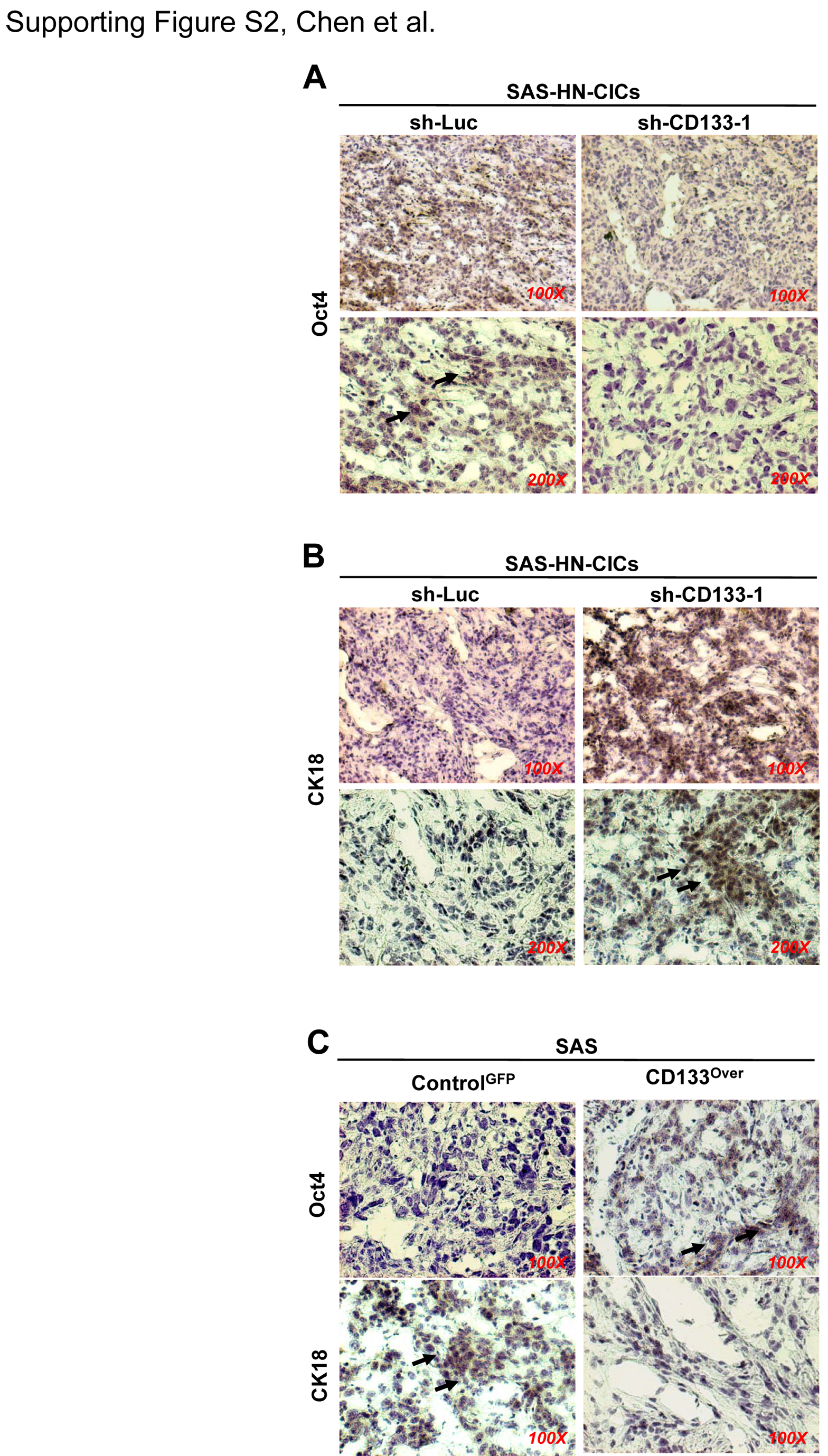

Supplement: Figure S2 — Expression of Oct4 and CK18 in tumors derived from CD133 ovexpressing HNSCCs or CD133 stable knockdown HN-CICs. Tumors derived from control HN-CICs (sh-Luc) or CD133-knockdown SAS-HN-CICs, and SAS cells were collected, sectioned and stained with Hematoxylin and anti-Oct4 (A) or anti-CK18 (B) as described. Arrows indicate the positive staining. (C) Tissue sections of control-GFP and CD133-overexpressing SAS cells xenograft tumor were stained with CK18 or Oct4. (TIF) [file pone.0028053.s002.tif]

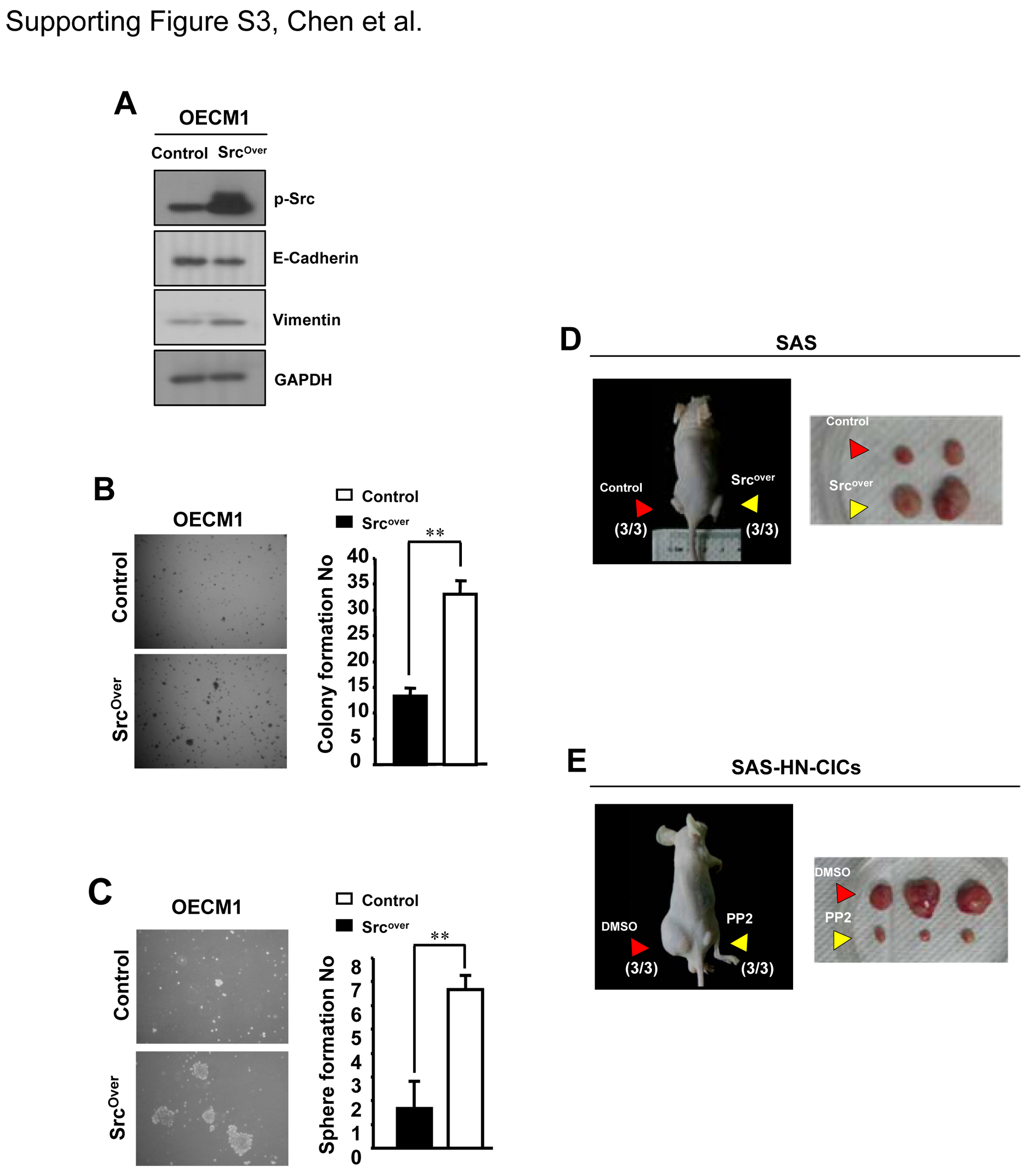

Supplement: Figure S3 — Src kinase activation promotes EMT, tumorigenicity and stemness. (A) Expression of p-Src, E-cadherin and Vimentin in HNSCCs transfected with either Src-overexpressing or control vector was examined by western blot. The amount of GAPDH protein was referred as loading control. (B) Anchorage-independent growth of control-vecor or Src-overexpressing HNSCCs was analyzed (**, p<0.01). (C) Sphere formation ability of control or Src-overexpressing HNSCCs was examined under the serum-free defined selection medium (**, p<0.01). (D) Representative tumor growth of control- or Src-overexpressing (Srcover) HNSCCs in the subcutaneous space of recipient mice (Red arrows: control HNSCCs; Yellow arrows: Src-overexpressing HNSCCs). (E) SAS HN-CICs (5*105 cells) were subcutaneously injected into both backs of nude mice and allowed to develop tumors to a size around 0.2 cm3 (12 days). On day 12, 15, and 18 after the inoculation of HN-CIC cells, PP2 (10 µM) was injected into the right back tumors whereas DMSO was injected into the left back as negaive control (Red arrows: DMSO treatment as control; Yellow arrows: PP2 treated HN-CICs). (TIF) [file pone.0028053.s003.tif]

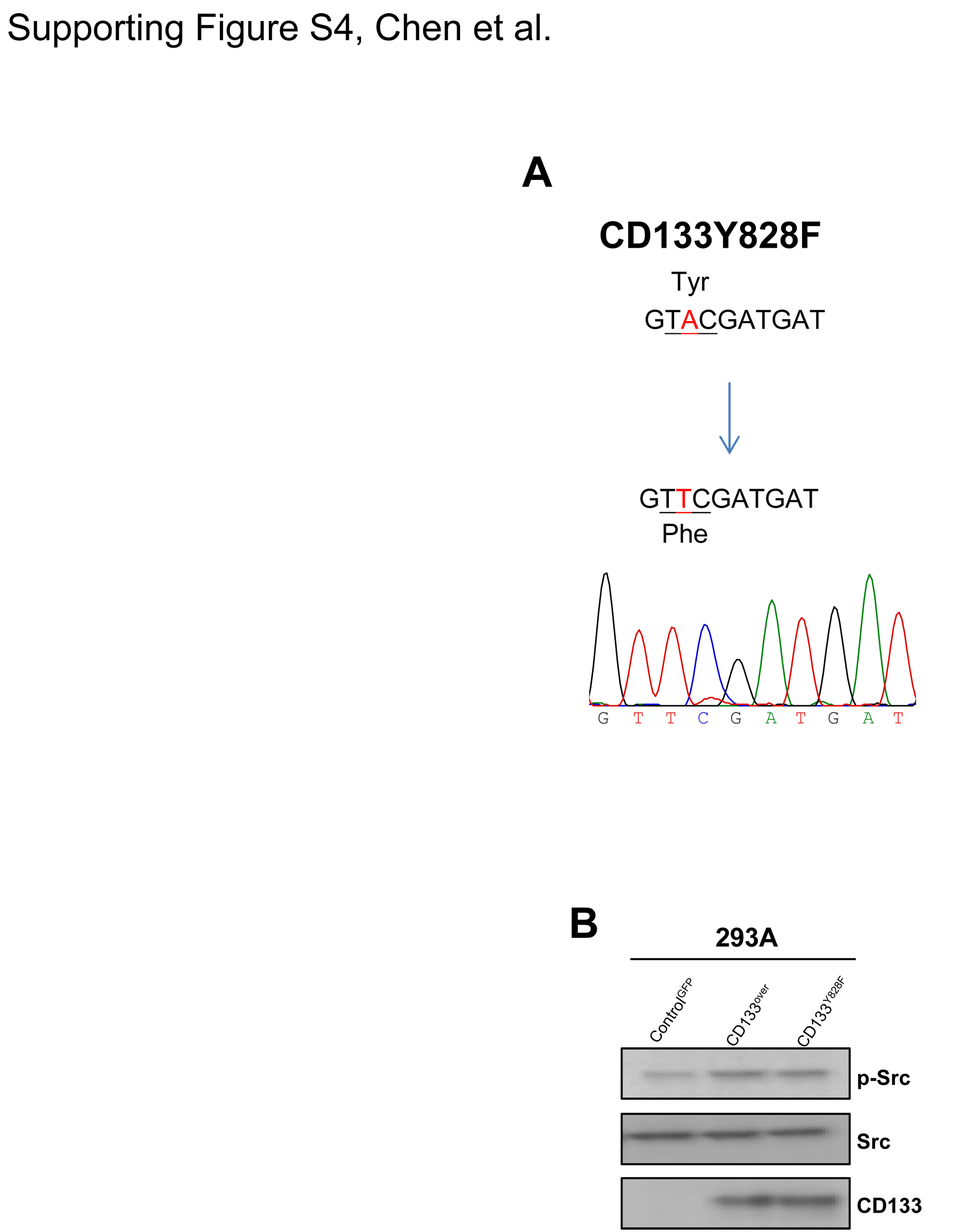

Supplement: Figure S4 — Effect of CD133Y828F mutant on p-Src activity. (A) DNA sequencing confirmed the CD133Y828F mutation, which encodes CD133Y828F mutant protein. (B) 293A cells transfected with plasmids, control-GFP, CD133 wild type (CD133over) or CD133Y828F (CD133Y828F), respectively, were collected (under no serum culture condition). The protein levels of p-Src, total Src and CD133 were examined by immunoblot. (TIF) [file pone.0028053.s004.tif]
